# Schwann cell myelination requires Dynein function

**DOI:** 10.1186/1749-8104-7-37

**Published:** 2012-11-20

**Authors:** Melissa M Langworthy, Bruce Appel

**Affiliations:** 1 Departments of Pediatrics and Cell and Developmental Biology, University of Colorado School of Medicine, MS 8108, Aurora, CO, 80045, USA

**Keywords:** Glia, Peripheral nerve, Dync1h1, Zebrafish, Myelin

## Abstract

**Background:**

Interaction of Schwann cells with axons triggers signal transduction that drives expression of Pou3f1 and Egr2 transcription factors, which in turn promote myelination. Signal transduction appears to be mediated, at least in part, by cyclic adenosine monophosphate (cAMP) because elevation of cAMP levels can stimulate myelination in the absence of axon contact. The mechanisms by which the myelinating signal is conveyed remain unclear.

**Results:**

By analyzing mutations that disrupt myelination in zebrafish, we learned that Dynein cytoplasmic 1 heavy chain 1 (Dync1h1), which functions as a motor for intracellular molecular trafficking, is required for peripheral myelination. In *dync1h1* mutants, Schwann cell progenitors migrated to peripheral nerves but then failed to express Pou3f1 and Egr2 or make myelin membrane. Genetic mosaic experiments revealed that robust Myelin Basic Protein expression required Dync1h1 function within both Schwann cells and axons. Finally, treatment of *dync1h1* mutants with a drug to elevate cAMP levels stimulated myelin gene expression.

**Conclusion:**

Dync1h1 is required for retrograde transport in axons and mutations of Dync1h1 have been implicated in axon disease. Our data now provide evidence that Dync1h1 is also required for efficient myelination of peripheral axons by Schwann cells, perhaps by facilitating signal transduction necessary for myelination.

## Background

Motor control and sensation require that nerve impulses are rapidly and efficiently transmitted over long distances. This is achieved by axons, which relay electrical signals between the central nervous system and peripheral muscles and sensory elements, and Schwann cells, which enhance the speed and efficiency of signal propagation by ensheathing peripheral axons with insulating myelin. Peripheral nerves can be very long, reaching more than 1 m in humans, but elaboration of a far-reaching peripheral nerve network comes with an apparent cost in that it is highly susceptible to disease. More than 100 kinds of peripheral neuropathy affecting motor, sensory and autonomic systems, and numerous degenerative diseases that attack, in particular, motor neurons, have been described.

Investigation of the molecular mechanisms of peripheral nerve disease has revealed that disruption of axon transport can cause nerve dysfunction and degeneration [1]. Molecular transport in healthy axons requires microtubules upon which cargo is carried, Kinesins, motors that generally move cargo from the neuron soma toward the axon tips, and Dynein cytoplasmic 1 heavy chain 1 (Dync1h1), which moves cargo in the opposite, retrograde direction. Disruption of any one of these components can cause disease. For example, a missense mutation that changes an amino acid within the homodimerization domain of Dync1h1 has been found in affected members of a family diagnosed with a dominant, axonal form of Charcot-Marie-Tooth (CMT) Disease [2] that is characterized by distal muscle weakness and atrophy, and mutations of the p150^Glued^ subunit of Dynactin, which interacts with Dync1h1, have been identified in families with slowly progressive lower motor neuron disease and amyotrophic lateral sclerosis [3,4]. Furthermore, dominant mutations of Dync1h1 that cause loss of proprioceptive sensory neurons have been identified in mice [5-7]. Whether disruption of Dynein-mediated molecular transport in other cellular components of peripheral nerves contributes to disease is not known.

Through a forward genetic approach using zebrafish, we found that Dync1h1 function is essential for peripheral myelination. Schwann cells were present at peripheral axons in *dync1h1* mutant larvae but did not wrap them with multiple layers of membrane or express myelin genes. Genetic mosaic experiments revealed that, in addition to its role in axon transport, Dync1h1 is required in Schwann cells for efficient myelination. Deficits in peripheral myelin were improved by stimulating cAMP level, which normally mediates axon signaling necessary for myelination, raising the possibility that transduction of the myelination signal requires Dync1h1-mediated molecular transport. These insights indicate that disruption of molecular transport mechanisms might contribute to peripheral disease by affecting both axons and Schwann cells.

## Results

### *dync1h1* function is essential for Schwann cell *myelin basic protein* expression

**Figure 1:**
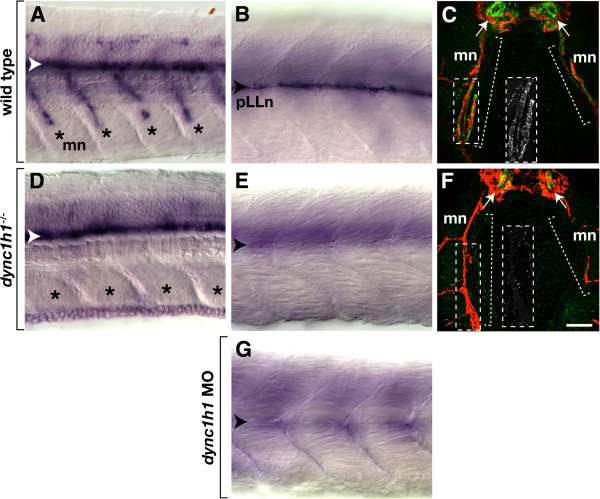
***dync1h1 *-deficient larvae express *mbp *RNA and protein in the central nervous system (CNS) but not the peripheral nervous system (PNS).** (**A**, **B**) *mbp* RNA expression in the ventral spinal cord (white arrowhead), along motor nerves (mn, asterisks) and at the posterior lateral line nerve (pLLn, black arrowhead) of wild-type larvae. (**C**) Immunohistochemistry reveals Mbp (green) associated with motor nerves marked by Acetylated Tubulin staining (red, brackets). Arrows indicate prominent Mbp in the ventral spinal cord. The inset shows Mbp expression, alone, of the portion of the motor nerve, indicated by the dashed box. (**D**, **E**) *mbp* RNA expression in the spinal cord but not at the motor nerves or the pLLn of *dync1h1* mutant larvae. (**F**) Mbp is absent from motor nerves and reduced in the ventral spinal cord of a mutant larva. The inset shows Mbp labeling, alone, of the portion of the motor nerve indicated by the dashed box. (**G**) pLLn of a wild-type larvae injected with *dync1h1* antisense MO lacks *mbp* RNA expression. Panels **A**, **B**, **D**, **E** and **G** show whole 4.25 days post fertilization (dpf) larvae at the level of the mid-trunk with anterior to the left and dorsal to the top. Panels **C** and **F** show transverse sections through the level of the trunk with dorsal up. Scale bars equal 20 μm (**A**, **B**, **D**, **E**, **G**), 40 μm (**C**, **F**) and 50 μm for the insets.

In a forward genetic screen for mutations that disrupt glial development, we identified an allele of *dync1h1* that caused both central nervous system (CNS) and peripheral nervous system (PNS) myelination defects. The CNS and PNS defects were distinct from each other, therefore, we report here only an investigation of the PNS phenotype, using the previously identified *dync1h1*^*hi3684Tg*^ mutant allele [8], which has a retroviral insertion at intron 52 [9]. The new allele and its associated CNS phenotype will be reported elsewhere. To assess myelination, we first used *in situ* hybridization to detect expression of *myelin basic protein* (*mbp*) RNA. At 4.25 days post fertilization (dpf), wild-type larvae expressed *mbp* RNA in the spinal cord and along motor roots (Figure 1A) and at the posterior lateral line nerve (pLLn) (Figure 1B). By 6 dpf, immunohistochemistry revealed Mbp associated with motor axons (Figure 1C) and the pLLn (data not shown). By contrast, *dync1h1* mutant larvae expressed *mbp* RNA within the spinal cord (Figure 1D) but not along motor roots or the pLLn (Figure 1D, E). Consistent with the reduction of *mbp* transcripts, very little Mbp was evident associated with peripheral axons in *dync1h1* mutant larvae (Figure 1F, data not shown). Most wild-type larvae (101 of 108), injected with antisense morpholino oligonucleotides (MO), which was designed to block translation of *dync1h1* mRNA [9], also lacked *mbp* expression (Figure 1G), confirming that the *mbp* expression defect of mutant larvae resulted from disruption of the *dync1h1* gene. Therefore, Dync1h1 function is essential for peripheral myelin gene expression.

**Figure 2:**
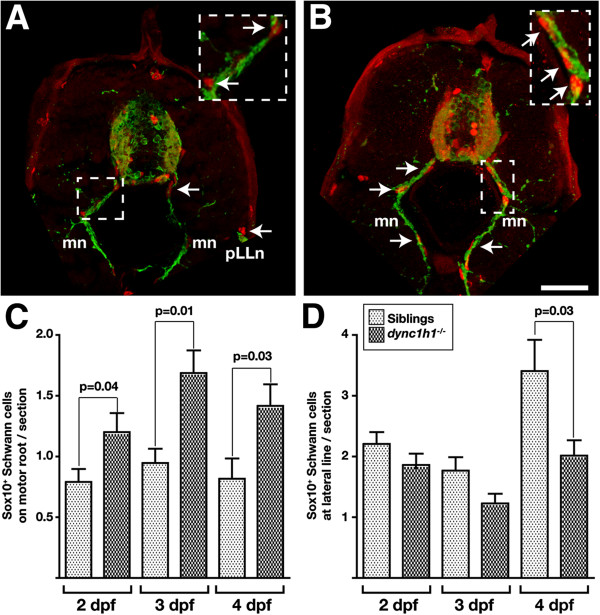
**Schwann cells are present at peripheral nerves in *dync1h1 *mutant larvae.** (**A**, **B**) Images of transverse sections at the level of the mid-trunk of 3 dpf wild-type (**A**) and *dync1h1* mutant (**B**) larvae labeled with antibodies to reveal Sox10 (red) and acetylated tubulin (green), which mark Schwann cells and axons, respectively. Schwann cells (arrows) are close to axons of motor nerves (mn) and the posterior lateral line nerve (pLLn). Insets are higher magnification views of motor nerves indicated by the dashed boxes. (**C**, **D**) Quantification of Schwann cells associated with motor roots (**C**) and pLLn (**D**). Significance of differences in Sox10^+^ Schwann cell numbers were calculated using nonparametric Mann–Whitney U analysis. Error bars represent standard error of the mean (SEM). Scale bar equals 80 μm for the main panel and 40 μm for the insets.

*dync1h1* mutant larvae might lack peripheral *mbp* expression because they lack Schwann cells or because Schwann cells fail to differentiate. To distinguish between these possibilities we examined Sox10 expression, which marks Schwann cell progenitors [10,11]. Similar to wild-type larvae, Sox10^+^ cells were close to both motor axons and pLLn axons in *dync1h1* mutant larvae (Figure 2A, B). In fact, more Sox10^+^ cells were associated with motor axons in *dync1h1* mutant larvae than wild type at 2, 3 and 4 dpf (Figure 2C). The number of Sox10^+^ cells at the pLLn did not differ between wild type and mutant at 2 and 3 dpf, however, Sox10^+^ cells did not increase in *dync1h1* mutant larvae between 3 and 4 dpf, resulting in a deficit relative to wild type (Figure 2D). We conclude that the absence of peripheral *mbp* expression in *dync1h1* mutant larvae does not result from an absence of Schwann cells. Therefore, lack of *mbp* expression is consistent with the possibility that Schwann cells fail to differentiate in mutant larvae.

**Figure 3:**
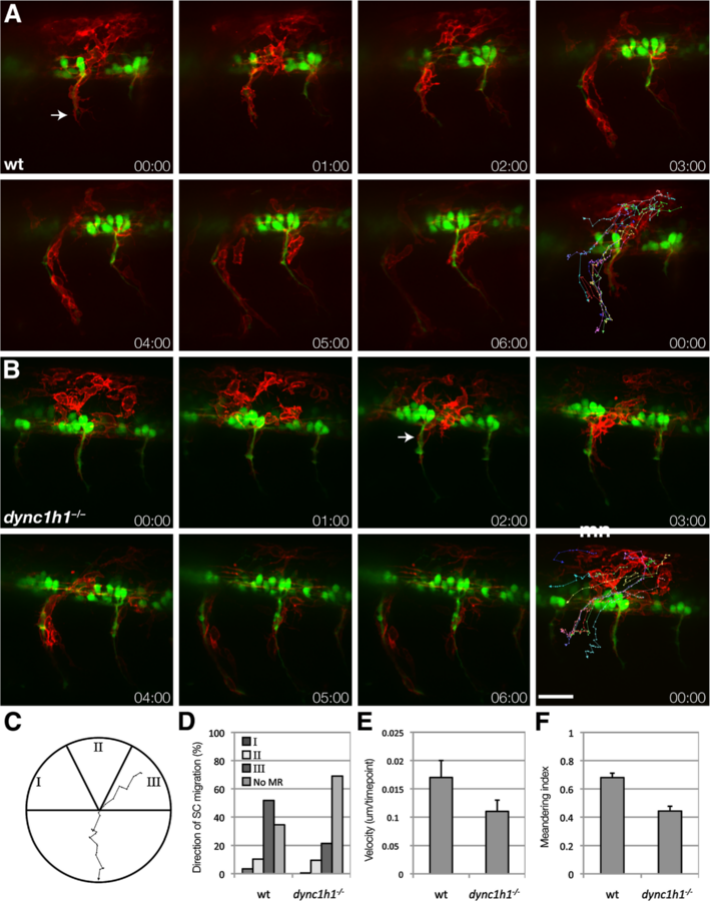
**Schwann cells migrate to motor axons in *dync1h1 *mutant larvae.** (**A**, **B**) Frames from time-lapse movies of wild-type (**A**) and *dync1h1* mutant (**B**) larvae carrying *Tg(sox10:mRFP)* and *Tg(mnx1:GFP)* transgenes to mark Schwann cells (red, arrow) and motor axons (green). Each sequence starts at 20 hours post fertilization (hpf) and elapsed time is shown in each panel. The final panel in each series shows tracks plotted for the migration of ten RFP^+^ Schwann cells over 6 hours, with 10 time points per hour. Images shown are lateral views of mid-trunk spinal cord with dorsal up. (**C**-**F**) Quantification of Schwann cell progenitor migration. The migration of ten *sox10*:mRFP^+^ Schwann cells were tracked over 6 hours with 10 time points per hour. (**C**) Schwann cell trajectories were assigned a direction of movement based on their starting and ending positions where the apex was assigned as the motor root exit point. I corresponds to anterior to posterior migration, II corresponds to dorsal to ventral migration, and III indicates posterior to anterior Schwann cell migration. A representative track from a wild-type embryo is displayed. (**D**) Quantification of Schwann cell trajectories from three embryos (30 Schwann cells) indicates that migration to the motor root is reduced but not eliminated in *dync1h1* mutants. Categories I, II and III correspond to the diagram in panel C. No MR represents the Schwann cells that did not reach the motor root. (**E**) Schwann cell velocity is reduced in *dync1h1*^*−/−*^ embryos (*P* = 0.004). (**F**) Schwann cell meandering index, calculated by the displacement from the origin by the track length, is reduced in *dync1h1*^*−/−*^ embryos (*P* < 0.0001) indicating less directed movement. *P*-values were calculated for each track using nonparametric Mann–Whitney *U*-test statistical analysis. Error bars represent standard error of the mean (SEM). Scale bar equals 40 μm.

We next examined the behavior of Schwann cell progenitors using time-lapse imaging. In wild-type *Tg(sox10:mRFP)* embryos, which express membrane-tethered RFP under control of *sox10* regulatory DNA [12], neural crest-derived Schwann cell progenitors converged near motor axon exit points (MEPs) and migrated in chain-like fashion along motor roots (Figure 3A). Schwann cell progenitors moved slower and less directly toward MEPs in *dync1h1* mutant embryos than in wild type (Figure 3B-F). Nevertheless, Schwann cell progenitors consistently arrived at and migrated along motor roots. We did not observe abnormal migration of Schwann cell progenitors at the pLLn (data not shown). These data show that the lack of *mbp* expression in *dync1h1* mutant larvae does not result from absence of Schwann cells. Instead, *dync1h1* function must be necessary for Schwann cell differentiation.

### *dync1h1* function is required for axon wrapping and myelination

**Figure 4:**
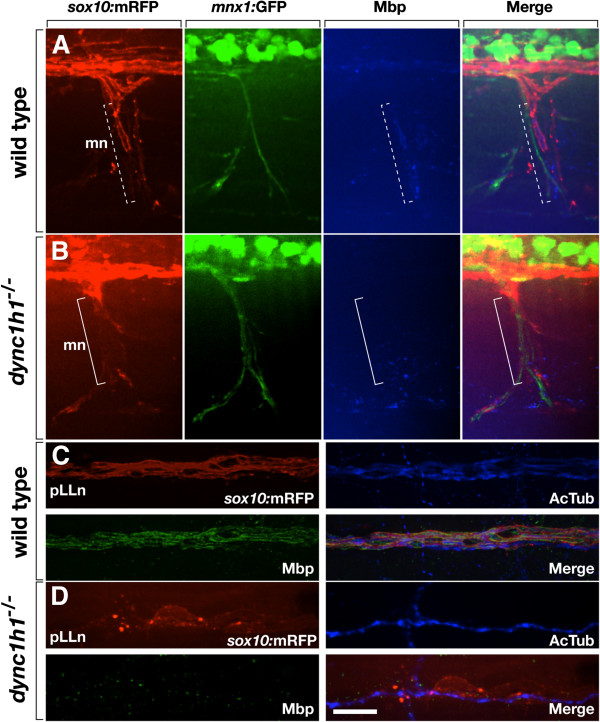
**Loss of *dync1h1 *function disrupts axon wrapping and Mbp expression by Schwann cells.** (**A**, **B**) Wild-type and *dync1h1* mutant larvae expressing *sox10:*mRFP (red) and *mnx1*:GFP (green) and labeled by anti-Mbp antibody (blue). Tube-like Mbp^+^ Schwann cells are evident at some axons of the motor nerve (mn) in wild type (dashed bracket) whereas Mbp^–^ Schwann cells appear more loosely associated with axons in the mutant (solid bracket). (**C**, **D**) Wild-type and *dync1h1* mutant larvae expressing *sox10:*mRFP (red) and labeled by anti-Mbp (green) and anti-acetylated tubulin (AcTub, blue) antibodies. Mbp^+^ Schwann cells ensheath pLLn axons in wild-type. By contrast, RFP^+^ Schwann cells associated with the pLLn in *dync1h1*^*−/−*^ mutants do not express Mbp and appear loosely associated with axons. All panels show lateral views of 6 dpf wild-type and *dync1h1*^*−/−*^ larvae with anterior to the left and dorsal to the top. Scale bars equal 20 μm (**A**, **B**) and 10 μm (**C**, **D**).

Although the above data show that Schwann cells were close to peripheral nerves in *dync1h1* mutant larvae it was not apparent whether they wrapped axons. We investigated the structure of peripheral nerves by examining Schwann cell morphologies revealed by transgenic reporter gene expression. Motor axons of wild-type larvae, marked by *Tg(mnx1:GFP)* expression [13], were ensheathed by tube-like *sox10:*mRFP^+^ Schwann cells, which expressed Mbp (Figure 4A). By contrast, Schwann cells with tube-like morphologies and Mbp expression were not evident in *dync1h1* mutant larvae and Schwann cells appeared to have loose contact with axons (Figure 4B). We also examined Schwann cells at the pLLn marked by *sox10:*mRFP expression in combination with immunohistochemistry to reveal axons. Whereas many tubular Mbp^+^ Schwann cells were evident at the pLLn of wild-type larvae (Figure 4C), in *dync1h1* mutant larvae Schwann cells, which did not express Mbp, appeared more loosely associated with the nerve (Figure 4D). Additionally, whereas motor axon bundles were similar in wild-type and mutant larvae (Figures 1C, F, 2A, B, 4A, B), the pLLn of mutant larvae appeared to have fewer axons than wild type (Figure 4C, D).

### Dync1h1 function within Schwann cells promotes myelination

**Figure 5:**
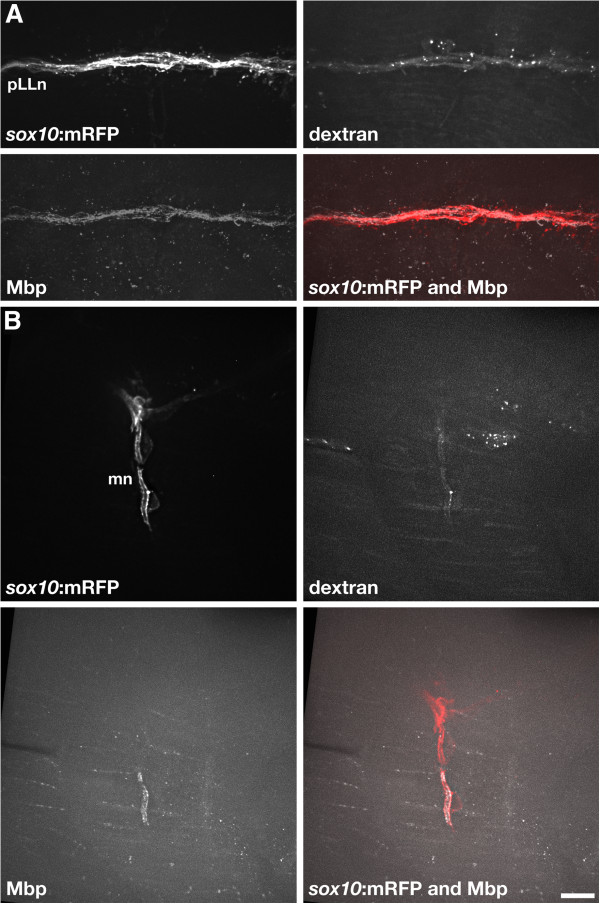
**Wild-type Schwann cells express Mbp when associated with *dync1h1 *mutant axons.** (**A**) Transplanted wild-type Alexa488-dextran^+^*sox10:*mRFP^+^ Schwann cells associated with pLLn axons of a 5 dpf *dync1h1* mutant host larva. The transplanted Schwann cells expressed Mbp, detected by immunohistochemistry. (**B**) Two transplanted wild-type Alexa488-dextran^+^*sox10:*mRFP^+^ Schwann cells associated with motor axons of a *dync1h1* mutant host larva. One of the two transplanted Schwann cells expressed Mbp. Scale bar equals 10 μm.

A failure of myelination in *dync1h1* mutant larvae could result from a defect in either axons or Schwann cells or in both axons and Schwann cells. For example, *dync1h1*^*−/−*^ axons might not provide a myelination signal to Schwann cells or mutant Schwann cells might not receive or transduce a myelination signal from axons. To distinguish between these possibilities we used cell transplantation to create genetic mosaic larvae. To investigate if wild-type Schwann cells can activate expression of Mbp when associated with *dync1h1* mutant neurons, we transplanted at blastula stage, cells from wild-type *Tg(sox10:mRFP)* embryos labeled with Alexa488-conjugated dextran, to nontransgenic embryos produced by matings of *dync1h1*^*+/−*^ adults. In this experiment the dextran label allowed us to select mutant host larvae that had peripheral nerves containing wild-type Schwann cells but not wild-type axons. These were then processed for immunohistochemistry to detect Mbp at 5 dpf. At both the pLLn (Figure 5A) and motor nerves (Figure 5B) of *dync1h1* mutant larvae, wild-type Schwann cells wrapped mutant axons normally and expressed Mbp (n = 9 and n = 14 larvae, respectively). We also performed the reciprocal experiment whereby we transplanted cells from blastula stage *Tg(sox10:mRFP)* embryos injected with *dync1h1* MO to wild-type embryos. Although we could identify transplanted cells within the premigratory neural crest population, we never found them associated with motor nerves during the normal period of myelination. We found a few transplanted, *dync1h1-*deficient cells at the pLLn, however, these had abnormal morphologies and occupied very narrow spaces between neighboring wild-type Schwann cells (data not shown). We speculate that *dync1h1*-deficient Schwann cells are at a competitive disadvantage for positions on peripheral axons when mixed with wild-type Schwann cells.

**Figure 6:**
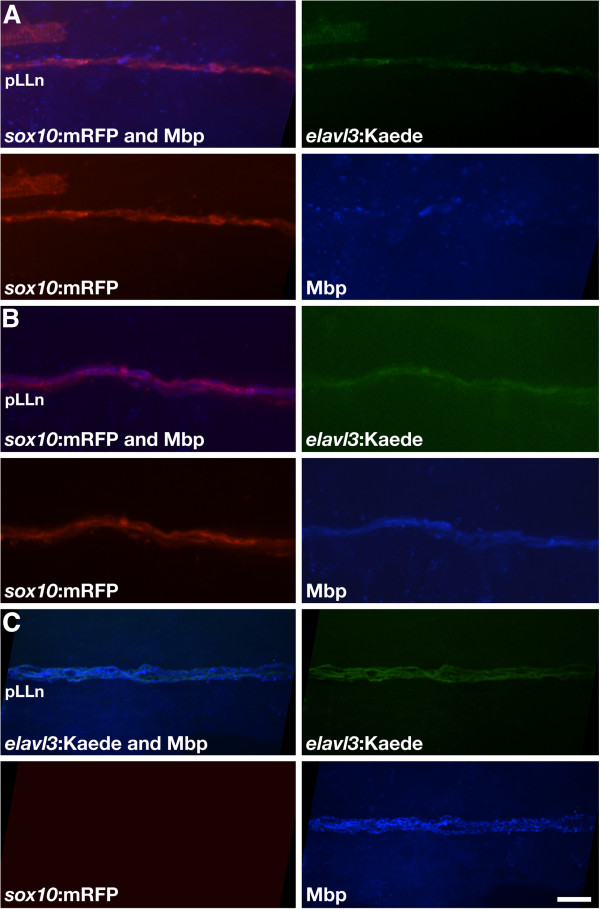
***dync1h1 *function is required in Schwann cells for robust Mbp expression.** (**A**) Example of *dync1h1*^*−/−*^*sox10:*mRFP^+^ Schwann cells (red) expressing Mbp (blue) at low or undetectable levels when associated with transplanted wild-type *elavl3:*Kaede^+^ pLLn axons (green). (**B**) Example of *dync1h1*^*−/−*^*sox10:*mRFP^+^ Schwann cells expressing detectable levels of Mbp when associated with transplanted wild-type *elavl3:*Kaede^+^ pLLn axons. (**C**) Example of transplanted wild-type Schwann cells expressing Mbp when associated with transplanted wild-type pLLn axons in a *dync1h1* mutant host larva. Scale bar equals 10 μm.

To investigate if mutant Schwann cells can express myelin in the presence of wild-type axons, we transplanted at blastula stage, cells from wild-type *Tg(elavl3:Kaede)* embryos, to embryos produced by matings of *Tg(sox10:mRFP)*;*dync1h1*^*+/−*^ adults. In this experiment, we could recognize any mutant host larvae with wild-type donor motor and pLLn axons labeled by *elavl3:*Kaede expression. We analyzed a total of 16 mutant host larvae that had wild-type *elav3:*Kaede^+^ pLLn axons associated with mutant *sox10*:mRFP^+^ Schwann cells. In 75% of these chimeric larvae (n = 12 of 16), mutant *sox10:*mRFP^+^ Schwann cells associated with wild-type axons had normal wrapping morphologies but apparently only low levels of Mbp, localized in a punctate pattern (Figure 6A). In the remaining 25% of these host larvae (n = 4 of 16), mutant Schwann cells associated with wild-type axons appeared to express and localize Mbp normally (Figure 6B). We also found two mutant host larvae that had pLL nerves consisting of both wild-type axons and wild-type Schwann cells, inferred by the absence of *sox10*:mRFP expression; these appeared to express Mbp normally (Figure 6C). Additionally, three mutant host larvae had mutant Schwann cells associated with motor nerves that contained wild-type axons; these Schwann cells neither wrapped axons nor expressed Mbp (data not shown). Taken together, our genetic mosaic data indicate that *dync1h1* functions in Schwann cells to promote myelination because wild-type Schwann cells can express Mbp when in contact with axons lacking *dync1h1*. However, they also indicate that the *dync1h1* is not absolutely required in Schwann cells for myelination because mutant Schwann cells can sometimes express Mbp when associated with wild-type axons. Therefore, efficient and robust myelination appears to require *dync1h1* function in both Schwann cells and neurons.

### *dync1h1* function is required for Schwann cell differentiation

**Figure 7:**
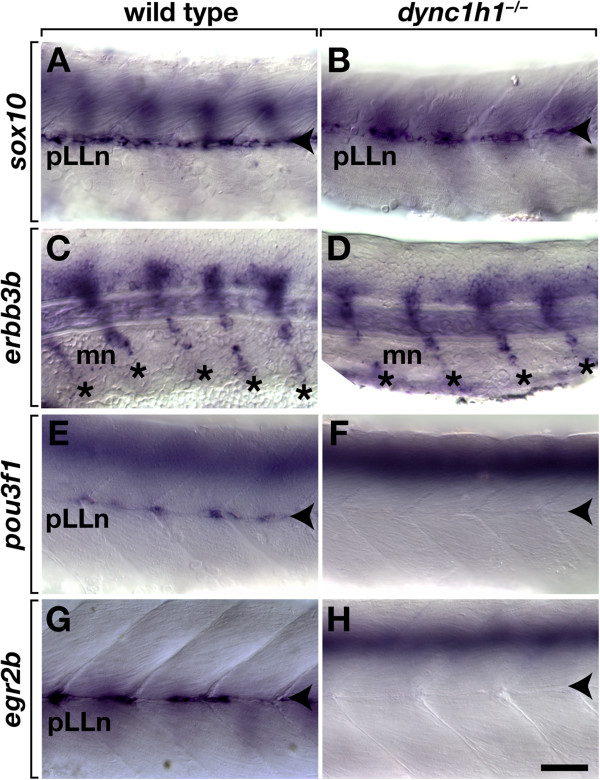
**Schwann cells require *dync1h1 *function to express transcription factors necessary for myelination.***sox10* expression is similar at the pLLn (arrowheads) in 52 hours post fertilization (hpf) wild-type (**A**) and *dync1h1* (**B**) mutant embryos. Schwann cells associated with motor nerves (mn, asterisks) express *erbb3b* in both wild-type siblings (**C**) and *dync1h1* mutant (**D**) embryos 52 hpf. Whereas 52 hpf wild-type embryos express *pou3f1* at the pLLn (**E**), expression is absent or present in only a few cells in *dync1h1* mutant embryos (**F**). At 72 hpf wild-type larva expresses *egr2b* at the pLLn (**G**), whereas comparably staged *dync1h1* mutant larva expresses *egr2b* in few or no cells (**H**). Images show lateral views of whole embryos and larvae focused on the mid trunk, with anterior to the left and dorsal up. Scale bar equals 40 μm.

Our data show that in the absence of *dync1h1* function, Schwann cell progenitors migrate to peripheral axons but fail to ensheath them normally or express a myelin gene. This raised the possibility that Schwann cells require *dync1h1* to progress from a progenitor state to a mature, myelinating state. To test this hypothesis, we investigated expression of genes that characterize distinct stages of Schwann cell differentiation [14]. Consistent with immunohistochemistry data described above, *sox10* RNA expression, which marks neural crest cells and Schwann cell progenitors, was similar in wild-type and *dync1h1* mutant larvae (Figure 7A, B). Although Schwann cells associated with the pLLn expressed *erbb3b*, which encodes a receptor necessary for Schwann cell development [15], only very weakly (data not shown), motor root Schwann cells expressed it robustly, with no difference evident between wild-type and *dync1h1* mutant embryos (Figure 7C, D). Expression of *foxd3* and *tfap2a*, which encode transcription factors that function in the early Schwann cell lineage [16,17], was also indistinguishable between wild type and mutant (data not shown). Therefore, *dync1h1* mutant embryos express at least some markers of immature Schwann cell progenitors normally. As Schwann cells progress from progenitor to promyelinating states they express *pou3f1,* also known as *Oct6* and *SCIP*[18-21]. In wild-type zebrafish embryos *pou3f1* expression was evident along the pLLn (Figure 7E). By contrast, mutant embryos (n = 22) and *dync1h1* MO-injected embryos (n = 26) consistently had few cells that expressed *pouf31* at the pLLn (Figure 7F and data not shown). Differentiating Schwann cells express *Egr2*, also known as *Krox20*, and *Egr2* function is required for myelination [22]. Wild-type zebrafish larvae expressed *egr2b* at both motor roots (data not shown) and the pLLn (Figure 7G). In *dync1h1* mutants (n = 30) and *dync1h1* MO-injected larvae (n = 109), *egr2b* expression was either absent or expressed in very few Schwann cells associated with the pLLn (Figure 7H and data not shown). By contrast, *egr2b* expression was not altered in the CNS of *dync1h1*^*−/−*^ mutants. These data indicate that *dync1h1* is required for Schwann cell progenitors to progress to a mature, myelinating state.

**Figure 8:**
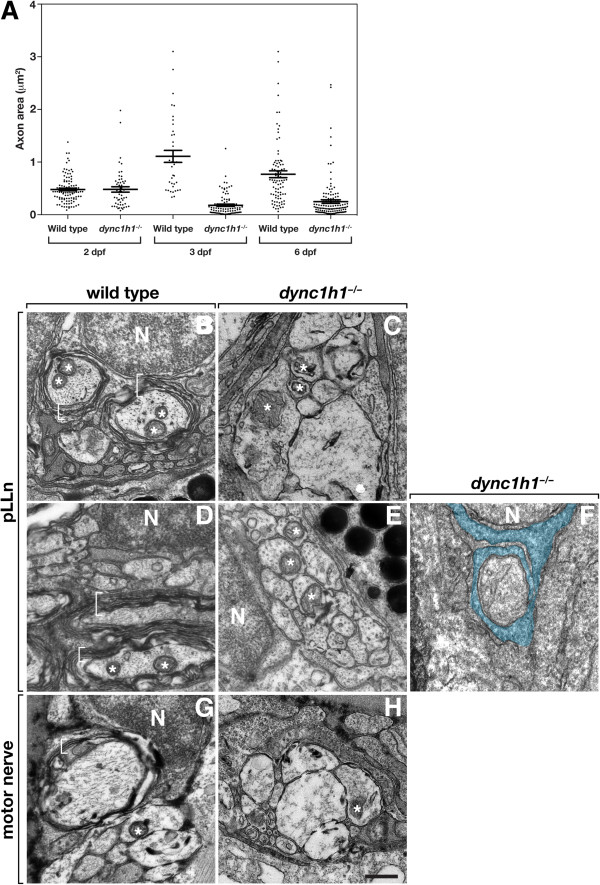
**Dync1h1 is required for the formation of myelinating Schwann cells.** (**A)** Scatter plot analysis of axon area. Each point represents one axon. Horizontal bars indicate mean axon area with SD for each group. Panels **B**-**F** show transmission electron microscopy (TEM) images of transverse sections through the pLLn, and panels **G** and **H** show coronal sections through motor nerves. At 3 days post fertilization (dpf), wild-type axons are loosely wrapped by multiple layers of myelin membrane (brackets) (**B**) whereas in a *dync1h1* mutant larva axons are not ensheathed by myelin (**C**). By 6 dpf myelin membrane is more compact in a wild-type larva (**D**) but still absent from a mutant larva (**E**). Panel **F** shows an axon wrapped by a single turn of loosely organized Schwann cell membrane (false colored blue). At 4 dpf, myelin ensheaths axon (bracket) of wild-type larvae (**G**) but is absent from motor axons of a *dync1h1* mutant larva (**H**). Asterisks mark mitochondria, which in mutant axons appear swollen and abnormally shaped. N, Schwann cell nucleus. Scale bar, 0.5 μm.

We next investigated the structure of peripheral nerves using transmission electron microscopy (TEM). At 2 dpf, *dync1h1* mutant embryos had an average of 5.3 axons within each pLLn (ten nerves in five embryos), whereas wild-type embryos had an average of 13.1 axons per pLLn (eight nerves in four embryos). Quantification of mean axon area and analysis of the distribution of axon area revealed no difference between mutant and wild type (Figure 8A). At 3 dpf, loosely wrapped myelin membrane was evident on some pLLn axons of wild-type larvae (Figure 8B). By contrast, pLLn axons of mutant larvae were devoid of myelin membrane (Figure 8C). The deficit of axons persisted, with an average of 14.8 axons per pLLn in mutants (six nerves in three larvae) and 36.6 axons per pLLn in wild type (six nerves in three larvae). Additionally, mean axon area was considerably smaller in mutant larvae (0.18 μm^2^) compared to wild-type (1.10 μm^2^). Scatter plot analysis revealed the presence of more very small axons in mutant compared to wild type (Figure 8A). By 6 dpf, the myelin membrane of wild-type larvae appeared more tightly compacted (Figure 8D) but the absence of myelin membrane on axons of mutant larvae persisted (Figure 8E). Although we could identify Schwann cells associated with the nerve, axons frequently did not appear to be wrapped by Schwann cell membrane (Figure 8C, E). However, some axons were wrapped by about 1.0 to 1.5 turns of Schwann cell membrane (Figure 8F). Again, the mean axon area of axons was smaller in mutants (0.24 μm^2^) than in wild type (0.76 μm^2^). Scatter plot analysis revealed that although *dync1h1* mutant larvae had some large diameter axons with the pLLn, the distribution was again skewed toward small diameter axons (Figure 8A). Additionally, we noted that mitochondria within mutant axons appeared swollen and irregularly shaped, which can be an indicator of axon degeneration and cell death [23]. We also investigated the structure of motor nerves at 4 dpf. Whereas loose myelin membrane was evident in wild-type larvae (Figure 8G), mutant motor nerves had no myelin and mitochondria appeared swollen (Figure 8H), similar to the pLLn.

### Elevating cAMP in *dync1h1* mutant larvae induces myelin gene expression

**Figure 9:**
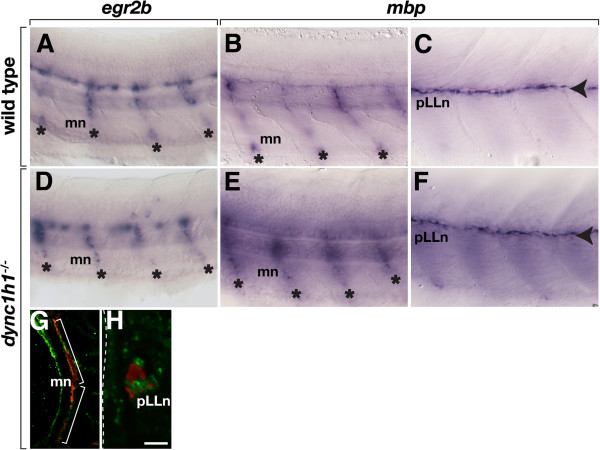
**Treatment with forskolin rescues peripheral myelin gene expression in *dync1h1 *mutant larvae.** Wild-type larvae treated with forskolin express *egr2b* (**A**) and *mbp* (**B**, **C**) RNA normally at motor nerves (mn, asterisks) and the pLLn (arrowhead). *dync1h1* mutant larvae treated with forskolin express *egr2b* (**D**) and *mbp* (**E**, **F**) RNA indistinguishably from wild type. In forskolin-treated *dync1h1*^*−/−*^ larvae, Schwann cells associated with motor nerve (**G**) and pLLn axons (**H**) marked by anti-acetylated tubulin staining (red), express Mbp (green). Images in **A**-**F** are whole mount views of 3 dpf larvae, anterior to the left and dorsal up. Images in **G** and **H** are single z-planes captured from transverse sections at 6 dpf. Dashed line indicates the lateral edge of the fish. Scale bars equal 20 μm (**A**-**F**), 40 μm (**G**) and 5 μm (**H**).

Treatment of Schwann cells cultured in the absence of axons with forskolin or cAMP analogs promotes myelin gene expression, indicating that an axon-derived myelination signal is transduced by an adenylyl cyclase/cAMP pathway [24]. Because Dync1h1 can promote signal transduction by transport of signaling endosomes [25], we investigated whether forskolin treatment of *dync1h1* mutant larvae could rescue myelin gene expression. Forskolin-treated *dync1h1* mutant larvae expressed *egr2b* RNA and *mbp* RNA at both motor nerves and the pLLn comparably to control and forskolin-treated wild-type larvae (Figure 9A-F). Immunohistochemistry revealed that Mbp expression was also restored at both motor nerves and the pLLn (Figure 9G, H). These data indicate that elevation of cAMP level can rescue the myelin gene expression deficit resulting from lack of *dync1h1* function, raising the possibility that Dync1h1 promotes myelination by promoting signal transduction necessary for myelination.

## Discussion

Dync1h1 is a subunit of the intracellular motor that transports cargoes on microtubules in a minus end-directed, or retrograde, direction. Cytoplasmic Dynein motors are crucial for a large variety of cellular functions [26,27], including transport of molecules within axons that promote neuron survival [25,28,29]. Accordingly, mutations that disrupt functions of Dynein motor subunits are implicated in axon disease [1]. Although all cells likely require cytoplasmic Dynein motor functions, in peripheral nerve disease disruption of Dynein-mediated transport has only been implicated to affect axons and not other cells that contribute to peripheral nerves, such as myelinating Schwann cells. Here we show that Dync1h1 function is essential for myelination of peripheral nerves in zebrafish.

Because cytoplasmic Dynein had not been previously implicated in peripheral myelination we were surprised to discover that *dync1h1* mutant zebrafish larvae did not express peripheral *mbp*, a myelin gene. Schwann cells were present at peripheral axons in mutant larvae, so the lack of *mbp* expression did not result from absence of Schwann cells. However, the Schwann cells did not wrap axons normally, sometimes ensheathing large diameter axons in only about one turn of Schwann cell membrane, and portions of some large diameter axons were not wrapped by any Schwann cell membrane. Schwann cells of mutant larvae also failed to express *pou3fI* and *egr2b*, which encode transcription factors essential for myelin gene expression. One interpretation of these data is that, in the absence of *dync1h1* function, Schwann cells fail to progress from a premyelinating, progenitor state to a fully differentiated, myelinating state.

Consistent with the known role of Dync1h1 in axon transport, peripheral axons in *dync1h1* mutant larvae had hallmarks of degeneration. Additionally, mutant larvae had fewer pLLn axons than wild type and mutant axons were, on average, smaller than wild type. Therefore, the failure of myelination could be an indirect consequence of an axonal defect. We attempted to test this possibility by creating different combinations of wild-type and mutant axons and Schwann cells. When we combined wild-type Schwann cells with mutant axons, the Schwann cells expressed Mbp indicating that Dync1h1 function is not required in axons for myelination. We also combined wild-type axons with mutant Schwann cells. In the majority of cases Mbp was either not evident or only present at very low levels. However, in 25% of the cases in which we analyzed wild-type pLLn axons, the associated mutant Schwann cells appeared to express Mbp at approximately normal levels. We interpret these data to mean that, although Schwann cells lacking Dync1h1 function can express Mbp when in the presence of wild-type axons, Dync1h1 in Schwann cells increases the efficiency and strength of Mbp expression.

If Dync1h1 function within Schwann cells enhances the efficiency of myelination, then stimulation of signaling pathways necessary for myelination might rescue the defects associated with the loss of Dync1h1 function. We tested this by treating mutant larvae with forskolin, which elevates cAMP levels. Whereas rat Schwann cells cultured with axons readily myelinate them, in the absence of axons Schwann cells do not express myelin genes. This requirement for axons can be circumvented by treating Schwann cells with forskolin or cAMP analogs [24]. Elevation of cAMP activates protein kinase A (PKA), and PKA inhibition interferes with myelination [30] indicating that PKA is a necessary component of the signal transduction pathway. A major target of the cAMP response is *Pou3f1/Oct6*[31], which may be activated following PKA phosphorylation of cAMP response element binding protein (CREB) and NF-κB transcription factors [32,33]. Pou3f1/Oct6 promotes transcription of *Egr2/Krox20*[34], which encodes a transcription factor that, with Sox10, drives expression of myelin genes [35-38]. Remarkably, forskolin fully restored *egr2b* and *mbp* expression in *dync1h1* mutant larvae, consistent with the possibility that Dync1h1 promotes myelination via a cAMP-dependent pathway.

How might Dync1h1 function in Schwann cells promote myelination? One possibility is that Dync1h1 is required for retrograde transport of signaling molecules from the Schwann cell-axon interface to the nucleus to regulate gene expression. Dynein-mediated retrograde transport of signaling endosomes along axons has been proposed to play key roles in neuron specification, axon outgrowth and neuron survival [25,28]. The best known example is internalization and trafficking of Trk receptors following neurotrophin stimulation [39-42] but signaling endosomes appear to contain entire signaling complexes with components of the Ras-MAP kinase, PLCγ and PI3 kinase pathways [39-41]. Transcription factors also might be included in signaling endosomes. CREB, translated from axon-localized mRNA in response to NGF, is transported to the cell nucleus to promote neuron survival [43] and nuclear translocation of NF-κB depends on Dynein function in various cell types [44]. Both CREB and NF-κB promote Schwann cell myelination [32,33], making them candidates for factors that that are actively transported within Schwann cells following activation of a cAMP-dependent signaling pathway.

Another possibility is that Dync1h1 is required for signaling by the G protein-coupled receptor (GPCR) Gpr126, which is essential for peripheral myelination in both zebrafish and mice [45,46]. Upon ligand binding, GPCRs stimulate activity of membrane-bound adenylyl cyclase via G proteins, to produce cAMP. Ligand binding also induces GPCR endocytosis and, although this is generally considered to be a mechanism for signal attenuation, in some circumstances cAMP production is enhanced by GPCR endocytosis [47,48]. Dynein is important for receptor sorting in early endosomes [49]. Together with the fact that forskolin treatment rescues the myelination defects of both *gp126*[45] and *dync1h1* mutant larvae, these observations raise the possibility that Dync1h1-mediated internalization and endosomal sorting of Gpr126 is essential for its signaling activity.

Finally, Dync1h1 might facilitate ErbB2/ErbB3 receptor signaling. ErbB receptors and the related EGF receptor are endocytosed upon ligand binding and trafficked through endosomes [50]. In fact, endocytosis and endosomal trafficking can transport ErbB2 to the nucleus of cultured cells [51] where, in principle, it could influence gene expression. Signaling mediated by ErbB2 and ErbB3 receptors promotes Schwann cell proliferation and migration [15]. We found that pLLn Schwann cells of *dync1h1* did not increase in number between 3 and 4 dpf as in wild type, and mutant Schwann cells also migrated more slowly and in less direct fashion. These apparently common roles open the possibility that Dync1h1-mediated endosomal trafficking influences ErbB receptor signaling.

## Conclusions

The work reported here shows that Dync1h1, a protein that is required for axon transport and implicated in diseases that affect peripheral axons, is necessary for myelination of peripheral axons by Schwann cells in zebrafish. Our genetic mosaic data provide evidence that efficient myelination requires Dync1h1 function in both Schwann cells and axons. Our observations raise the possibility that mutations of Dync1h1 cause nerve disease, not only by causing damage to axons but also by disrupting formation or maintenance of myelin.

## Methods

### Zebrafish husbandry

The University of Colorado Denver Institutional Animal Care and Use Committee approved all zebrafish studies. Zebrafish stains used include *dync1h1*^*hi3684Tg*^[8] (Amsterdam *et al*., [2005]), *Tg(mnx1:GFP)*^*ml2*^[13], *Tg(sox10:mRFP)*^*vu234*^[12], *Tg(elavl3:Kaede)*^*rw0130a*^[52] and AB. Embryos produced by paired matings were raised at 28.5°C, maintained in egg water or embryo medium, and staged according to hpf or dpf. Homozygous mutants for the *dync1h1*^*hi3684Tg*^ allele were created by pair-wise crossings of *dync1h1*^*hi3684Tg+/−*^ adults. Embryos younger than 48 hpf were genotyped using Hi3684_5E01: 5^1^-AAACCTACAGGTGGGGTCTTTC-3^1^ and Hi3684_5E02: 5^1^-GCTACAACTACGAGCAAGTCAACC-3^1^ as primers for PCR to amplify the mutant *dync1h1*^*hi3684Tg*^ allele (protocol available at Zebrafish International Resource Center). A second primer set 5^1^-TCTTTAGCGTCGTCCTCCAG-3^1^ and Hi3684_5E02 was used to amplify the wild-type allele.

### In situ RNA hybridization

Experiments were performed as described previously [53]. Probes used include *sox10*[10], *erbb3*[15], *mbp*[54], *egr2b*[55] and *pou3f1*[56]. Following *in situ* hybridization, tissues were fixed with 4% paraformaldehyde, equilibrated in 70% glycerol and mounted on glass coverslips for whole-mount imaging. Images were collected using a Zeiss Axio Observer equipped with DIC optics, Retiga Exi digital color camera and Volocity software (Improvision/PerkinElmer). All images were imported into Adobe Photoshop software and image processing was limited to changes in resolution, levels, contrast, brightness and cropping.

### Immunohistochemistry

Embryos and larvae were fixed in 4% paraformaldehyde, 8% sucrose, 1X PBS overnight at 4°C. For whole-mount immunocytochemistry, embryos and larvae were incubated in ddH_2_O for 4 hours, blocked in PBS-TX (1X PBS, 1% Triton X) containing 10% sheep serum and 10% BSA for 1 hour at room temperature (RT) and incubated in primary antibody diluted in antibody solution (2% sheep serum, 2% BSA, PBS-TX) for 24 hours at 4°C. After washing several hours in PBS-TX, secondary antibodies diluted in antibody solution were applied for 24 hours at 4°C. Embryos and larvae were washed in PBS-TX for at least 4 hours. For immunocytochemistry on sections, embryos and larvae were embedded in 1.5% agar/5% sucrose, frozen with 2-methyl-butane chilled by immersion in liquid nitrogen, and sectioned using a cryostat microtome (20 μm). Sections were re-hydrated with 1 × PBS and pre-blocked for 30 minutes in 2% sheep serum/BSA-1 × PBS. The sections were incubated with primary antibody overnight at 4°C, washed extensively with 1 × PBS and incubated with the appropriate fluorescent secondary antibody for 2 hours at RT. Once the secondary antibody was washed off, sections were covered with Vectashield (Vector Laboratories, Burlingame, CA, USA). Primary antibodies used were rabbit anti-Sox10 (1:200) [57], mouse anti-acetylated tubulin (1:5000, catalog T7451, Sigma-Aldrich, St. Louis, MO, USA) and rabbit anti-Mbp (1:200) [12]. Secondary antibodies used were Alexa Fluor 405-, 488- and 568-conjugated goat anti-rabbit; Alexa Fluor 405- and 568-conjugated goat anti-mouse (1:200, Life Technologies, Carlsbad, CA, USA).

### Transmission electron microscopy

A t 3, 4, or 6 dpf larvae were anesthetized with Tricaine, placed on ice, and fixed in a solution of 2% glutaraldehyde, 4% paraformaldehyde and 0.1 M sodium cacodylate, pH 7.4. Fixation was accelerated using a Biowave Pro Laboratory Microwave with ColdSpot (Ted Pella, Inc, Redding, CA, USA) maintained at 15°C. Membranes were enhanced using either secondary fixation with OsO_4_, uranyl acetate, and imidazole [15], or secondary fixation using OsO_4_-TCH-OsO_4_. Electron micrographs were collected using a FEI Techai G2 BioTwin microscope, transferred to Adobe Photoshop and image processing was limited to resolution, contrast, and cropping. The axon area and the length of the longest axis of each pLLn axon was measured using Volocity software.

### Forskolin treatment

Embryos were dechorionated and incubated in embryo medium containing 50 μM forskolin (F6886, Sigma) dissolved in dimethyl sulfoxide (DMSO). Control embryos were treated with an equal concentration of DMSO (0.1% in embryo medium). Treatments were from 45 to 52 hpf. Following treatment, embryos were placed in embryo medium without drug, fixed at the indicated times, and processed for *in situ* RNA hybridization or immunohistochemistry.

### Genetic mosaic analysis

Donor embryos were prepared by pair-wise crossing of either AB or *Tg(sox10:mRFP)* adults. Host embryos were prepared by pair-wise crossing of either *dync1h1*^+/−^ or *dync1h1*^+/−*;*^*Tg(mnx1:GFP)* adults. Donor embryos and host embryos at blastula stage were positioned into the molded wells of an agarose plate. Transplantation was performed as previously described [58] with approximately 20 donor cells transplanted into the host embryo. Wild-type *Tg(sox10:mrfp)* donors were injected with Alexa488-conjugated 10,000 MW dextran (Invitrogen) at cleavage stage to enable discrimination between donor and host cells. Alternatively, wild-type *Tg(elavl3:Kaede)* donors were transplanted to *Tg(sox10:mRFP);dync1h1*^*−/−*^ hosts. At 5 dpf, genetic mosaic larvae were fixed, processed for whole-mount immunocytochemistry, and imaged as described above. Larvae with Alexa488 dextran staining in the cell bodies of pLLn neurons or in spinal cord motor neurons, which could contribute wild-type axons to peripheral nerves, were removed from the study and not analyzed for expression of Mbp.

### Morpholino injections

We purchased a previously described antisense Morpholino oligonucleotide (MO) designed to block translation of *dync1h1* and consisting of the sequence CGCCGCTGTCAGACATTTCCTACAC [9] from Gene Tools, LLC (Corvallis, OR, USA. The MO was dissolved in water and diluted prior to injections. We injected 1 to 2 nL into the yolk just below the single cell of fertilized embryos. All MO-injected embryos were raised in embryo medium at 28.5°C.

### Time-lapse imaging

At 16 hpf, embryos were embedded in low melting point agarose and mounted in a heated chamber (28.5°C) of a motorized stage. Z-stack images were obtained every 10 minutes from 16 to 30 hpf using a PerkinElmer UltraVIEW VoX Confocal System coupled with a Zeiss Axio Observer inverted compound microscope fitted with a 40X oil immersion objective (NA = 1.3). Using Volocity software (PerkinElmer, Waltham, MA, USA), images were processed using deconvolution and contrast enhancement. Four-dimensional volumes were assembled at a speed of 6 to 8 time points/second and exported as QuickTime movie files.

## Abbreviations

cAMP: cyclic adenosine monophosphate; CREB: cAMP response element binding protein; DMSO: Dimethyl sulfoxide; Dpf: Days post fertilization; Dync1h1: Dynein cytoplasmic 1 heavy chain 1; CMT: Charcot-Marie-Tooth; CNS: Central nervous system; GPCR: G protein-coupled receptor; Hpf: Hours post fertilization; Mbp: Myelin basic protein; MEP: Motor axon exit point; MO: Morpholino oligonucleotide; PBS: Phosphate-buffered saline; PCR: Polymerase chain reaction; PKA: Protein kinase A; pLLn: Posterior lateral line nerve; PNS: Peripheral nervous system; RT: Room temperature; SEM: Standard error of the mean; TEM: Transmission electron microscopy; PNS: Peripheral nervous system.

## Competing interests

The authors declare that they have no competing interests.

## Authors’ contributions

MML performed all the experiments described in this manuscript. BA helped design and interpret experiments and wrote the manuscript with MML. Both authors read and approved the final manuscript.
